# A secure procedure for early career scientists to report apparent misconduct

**DOI:** 10.1186/s40504-020-00110-6

**Published:** 2021-01-25

**Authors:** Baruch Fischhoff, Barry Dewitt, Nils-Eric Sahlin, Alex Davis

**Affiliations:** 1 Department of Engineering & Public Policy, Carnegie Mellon University, Pittsburgh, PA USA; 2 Institute for Politics & Strategy, Carnegie Mellon University, Pittsburgh, PA USA; 3 Medical Ethics, Department of Clinical Sciences, Lund University, Lund, Sweden

**Keywords:** Research integrity, Scientific practice, Institutional design, Policymaking

## Abstract

Early career scientists sometimes observe senior scientists engage in apparent scientific misconduct, but feel powerless to intervene, lest they imperil their careers. We propose a Secure Reporting Procedure that both protects them, when pursuing those concerns, and treats the senior scientists fairly. The proposed procedure is, we argue, consistent with the ethical principles of the scientific community, as expressed in the codes of its professional organizations. However, its implementation will require changes in procedures and regulations. Those efforts will be a small price to pay for protecting the scientific community’s integrity and fidelity to its principles. We begin by describing the circumstances motivating the proposal, then sketch its design, and, finally, illustrate next steps in its application in two national settings.

In successful apprenticeships, early career scientists observe their mentors solving problems that they themselves will face, such as which studies to cite, what methods to use, and how to report results responsibly.

In unsuccessful apprenticeships, early career scientists may observe their mentors fabricating data or falsifying results, for example by massaging observations until they yield desired patterns. Such fabrication and falsification appear to account for some of the recently documented failures to replicate published findings (Yong 2018; Camerer et al. 2018).

When early career scientists observe such misconduct, they face a difficult choice. If they say nothing, they compromise their faith in science and their own ethical codes and, possibly, institutional ones. If they say anything, they incriminate people they know, and perhaps once respected, and risk retribution that may imperil their careers.

Complicating matters, early career scientists may be unsure whether their mentors are sloppy or dishonest. They may also wonder what responsibility they bear for a field that has normalized pathology, such that “everybody” excludes bothersome data as “noise.”

Early career scientists need strong support from their mentors, both overt (e.g., co-authorships, letters of recommendation) and covert (e.g., off-the-record opinions, networking, gossip). Raising questions of research integrity may not only forfeit that support, but induce the opposite, with vindictive mentors undermining their reputation and self-confidence.

We propose a reporting procedure designed to reduce the expected costs and increase the expected benefits for defending scientific integrity. Although it could be used by scientists at any career stage, it is meant to serve the most vulnerable: early career scientists working for senior ones, as students, post-docs, or junior faculty in departments with dominating personalities.

We recognize that implementing our proposal may entail a major undertaking, requiring negotiation of local norms and rules, even possibly the rewriting of laws. However, we believe that it addresses a structural problem in scientific institutions that existing procedures (e.g., an ombudsperson) cannot remedy. We believe that it provides an orderly, mutually respectful approach to a long-standing problem that could otherwise explode, in the current age of reckoning. We return to these concerns after presenting our proposal.

## The proposal

Our proposal is predicated on early career scientists having sovereignty over their actions. Thus, while it creates more favorable conditions for action, it does not force their hand in requiring it, respecting early scientists' right to control as much of an unhappy situation as possible. It recognizes that they may be:

- unsure how to interpret the behavior that they have observed;
- reluctant to discuss their experiences with others, not knowing how word will travel; and
- fearful of acting alone, lest they bear unacceptable career and personal risks.

As a result, action may only be possible if the senior scientist is completely discredited, reducing the risk of effective retaliation.

Our proposal also recognizes the rights of senior scientists to be protected against unfounded, and perhaps malicious charges. It has four stages:

### Individual submission

Early career scientists who have observed apparent misconduct file a confidential report with their institution’s *scientific integrity official*. That official helps them to interpret their experience, provides emotional support, and describes their options. If they choose to proceed on their own, they will be referred to the proper channels. If they wish to proceed, *but not alone*, they can file a report that remains entirely under their control. If they choose to end the inquiry, all records are erased.

### Report monitoring

The scientific integrity official monitors reports, looking for patterns of abuse associated with particular scientists or labs. If a pattern emerges, the official contacts each person who has filed a relevant report. The official describes the existence of other reports but says nothing about their contents or sources. The official asks filers which elements of their reports, if any, can be shared with other filers.

### Report coordination

The official shares information consistent with filers’ instructions. That sharing might entail anything from revealing no report details to convening a joint meeting. The official documents the process, including when sharing has breached the independence of the evidence. The official informs those who have not joined the process about its progress, while protecting the confidentiality of those who have joined.

### Action

Should some, or all, of the filers choose to proceed, the official informs them of their options and facilitates transferring the case to the proper jurisdiction. In order to ensure fairness to all parties, including the right to self-defense against charges, no action can be taken when senior scientists are deceased or incapacitated.

This procedure seeks to make joint action feasible, while preserving filers’ control over their own involvement. It may lead to *acceptable inaction*, if filers come to see the apparent misconduct more charitably. It might lead to *bitter inaction*, if filers conclude that they cannot take the risk of pursuing legitimate concerns. It might lead to *joint action*, if a safe forum exists. It might lead to *individual action*, if a filer is ready to risk having to leave the field.

## Implementation

The feasibility of this process depends, critically, on having scientific integrity officials with the wisdom to identify misconduct, distinguish honest mistakes, and detect unfounded accusations. These officials will also need the interpersonal skills and fortitude to counsel early career scientists who feel mistreated and senior scientists hoping to make amends. They might be retired scientists, who are experienced researchers, familiar with their institution’s ways and eager to protect its integrity.

Thus, our proposal protects early career scientists, by helping them to articulate their concerns and choose the best course of action (including, possibly, inaction). It protects senior scientists by helping early career scientists to assess the legitimacy of their concerns and express them professionally. It protects the scientific community by providing an orderly outlet for issues that would damage its reputation even more, were they to emerge chaotically.

Our proposal differs from some existing institutional procedures by not requiring action, even if both early career scientists and scientific integrity officers believe that there has been misconduct. It differs from some existing procedures by coordinating individual and collective action. It differs from still others by relying on moral sanctions, from disclosure, rather than legal or administrative ones.

Ethically, it puts the wellbeing of individual early career scientists above the accuracy of the scientific record, which public reports could correct. Pragmatically, it protects the scientific enterprise, by deterring future misconduct. Socially, it affirms the culture of science as a self-policing community, vulnerable to the subversion that our proposed procedure addresses.

We believe that our proposal captures the spirit of existing institutional and professional codes (Center for the Study of Ethics in the Professions n.d.; All European Academies 2017; National Academy of Sciences 2018). It provides a way to mitigate the power imbalances that limit the reporting of research misconduct, as identified in the recent National Academies of Sciences, Engineering, and Medicine consensus report on *Fostering Research Integrity* (NASEM 2017; also Kaplan 2019; Isai 2019; Farrow 2019; Peters 2019). That report explicitly recommends changing regulations that limit effective policies. It also advocates international collaboration in developing and implementing best practices. It recognizes the institutional inertia that such reforms will have to overcome. We propose a way to begin that hard work.

In the United States, one potentially needed regulatory change is allowing scientific integrity officials to protect the confidentiality of individual reports of potential misconduct. The administrators who determine legal reporting requirements would have to see greater value in having a few strong cases, which send signals through the scientific community, than in having many weak ones, perhaps leading nowhere. The challenges of changing reporting requirements are no excuse for perpetuating an ineffective system.

As an example of a step in this direction, a recent Swedish government report recommended changing the locus of investigations for serious research misconduct (falsification, fabrication, and plagiarism) from individual institutions to a national body (Fahlgren 2017). That report notes the importance of maintaining secrecy for both the accuser and accused, in much the spirit of our proposal.

We envision the procedure being implemented by individual academic institutions, as part of their commitment to providing a safe work environment, as well as protecting their reputation. However, the specifics of that implementation will depend on local constraints. For example, reporting requirements might lead to situating an institution’s scientific integrity office outside its legal bounds, as with the proposed Swedish national body. An institution might choose to impose the procedure or to develop it in consultation with its scientists, including perhaps some with something to hide. These potential barriers deserve the same creative problem solving as other pernicious problems, such as reporting sexual misconduct (“Project Callisto” 2021)*.*

The scientific community has always policed itself. There are now concerted efforts to improve that practice with procedures, such as pre-registering studies and analyses, so that everything gets reported, and archiving data, so that anyone can check the work. Those efforts have raised the status of “non-results,” so that researchers get credit for studies that fail to replicate earlier results or affirm plausible hypotheses (Kupferschmidt 2018; Nosek et al. 2018; Open Science Collaboration 2015).

Nonetheless, such voluntary efforts, and the social norms that they promote, will not deter some scientists, desperate for survival or acclaim. Our proposal is meant to demonstrate the scientific community’s commitment to honor scientists who maintain the integrity of the practices that differentiate science from other sources of claims about the world and to punish scientists who intentionally degrade those practices.

Our proposed procedure would help early career scientists find justice and peace, whether they choose to go public or just draw strength from having their concerns legitimated and heard (Flaherty 2019)*.* It would reduce the power imbalances that limit the scientific community’s progress. It would provide senior scientists with a measured, mediated process, focused on setting the research record straight. It would help defenders of the scientific community, by showing that scientists conscientiously look for problems, rather than begrudgingly admit them. It would strengthen the self-correcting processes that are essential to the progress of science – and that distinguish it from its critics.

## Data Availability

Not applicable.
